# 

**Published:** 1915-07-24

**Authors:** 


### Medical Appointments.

Mr. J. W. Watthews, M.B., Ch.B. Edin., has been appointed house surgeon to the Bradford Royal Infirmary.

The appointment is notified of Mr. J. W. Simon, M.R.C.S., M.R.C.P. (Lond.), as assistant medical officer to the Edmonton Military Hospital. The salary attached is to be on the usual Army scale.

### The Book Market.

LIST OF NEW BOOKS RECEIVED FROM THE FOLLOWING PUBLISHER:—

H. K. Lewis: "Amœbiasis and the Dysenteries." By L. P. Phillips, M.A., M.D. 6s. 6d. net. "Materia Medica and Pharmacy." Third edition. By Reginald R. Bennett, B.Sc. 4s. 6d. net. "War Surgery." By E. Delorme. Translated by H. de Meric. 5s. net.

### Dispensing for Women.

### JACOB BELL MEMORIAL SCHOLARSHIP.

Miss Dorothy Alice Bills, who was recently awarded a scholarship, is the first woman student to win the above prize since its inauguration.

Two scholarships are awarded annually of the value of £25 each, and the second winner for this year is Mr. David Page. Miss Bills served her apprenticeship with her father, Mr. A. J. Bills, of Kinver, Stourbridge, and Mr. Page, who was educated at the Alderman Newton School, Leicester, served his apprenticeship with Mr. Ward, of Leicester. The scholarship is tenable for one year, and the Council of the Pharmaceutical Society provides free laboratory instruction, thus enhancing the pecuniary value of the scholarships by about £40. In addition, books to the value of £5 are divided between the scholars. Full particulars of the scholarship and the regulations will be found in the Calendar of the Pharmaceutical Society, and a complete list of scholars since the year 1861. In this connection it is interesting to note that "Pharmacy and Dispensing for Nurses" is the title of a well-known book in the series published by The Scientific Press.

### NEW ISOLATION HOSPITAL AT THORPE.

The new Infectious Diseases Hospital built by the Easington Rural District Council at Thorpe, near Easington, at a cost of £12,600, has been formally opened. The architect was Mr. Hugh T. D. Hedley, of Sunderland, and the contractors were Messrs. Christopher Brown, Ltd., of West Hartlepool.

### Latest Installations in Hospital Equipment.

### WHAT OTHERS ARE DOING.

*** **We desire information calculated to keep this column up to date in the interest of institutional workers generally. Secretaries and other executive officers are invited to forward particulars of new installations in any department. In this way a file will be available for all hospital authorities contemplating improvements and anxious to know what other institutions have found effective and modern.**

*Double Windows*:

The Empire Hospital, Vincent Square, W., is fitting a series of inner casement windows. These are manufactured by Messrs. Holloway Bros. (London), Ltd., South Audley Street, W.

*Invalid Lifters*:

The Lewisham Military Hospital has installed a bedstead and Skeffington lifter combined, together with a Sacrum lifter. The maker is Arthur Skeffington, 49 Ulundi Road, Blackheath, S.E.

*Ironing Machine*:

The Guest Hospital, Dudley, is contracting for the supply of new laundry machinery (including washing machine, hydro extractor, Decoudun ironing machine, and collar-ironing machine) with the Cherry Tree Machine Co., Ltd., laundry engineers, Blackburn.

*Sterilisers*:

The Empire Hospital, Vincent Square, W., is installing a new patent high-pressure steriliser, manufactured by Jackson and Co., of Liverpool. This is being supplied by Cory Brothers, 54 Mortimer Street, London.

### Personalia.

A MANCHESTER SURGEON'S APPOINTMENT.

Mr. William Thorburn, of Manchester, has been appointed one of the consulting surgeons to the Mediterranean Expeditionary Force, and will shortly proceed to Malta to take up his duties. Mr. Thorburn has been Professor of Clinical Surgery at Manchester University since 1910, and is a member of the Council and of the Court of Examiners of the Royal College of Surgeons. He is also honorary surgeon to the Manchester Royal Infirmary, a lieutenant-colonel in the Royal Army Medical Corps (Territorials), and a referee under the Workmen's Compensation Act. His youngest and only surviving son was killed in action in the Dardanelles last month.

Rates of Subscription : Twelve months, 6/6 ; Six months, 4/- ; Three months, 2/-, post free to any address in the United Kingdom. Abroad, Twelve months, 8/8; Six months, 5/-; Three months, 2/6, post free.—Address The Subscription Dept., "The Hospital," The Hospital Building, 28/29 Southampton Street, Strand, London, W.C.

**28/29 SOUTHAMPTON STREET, STRAND, LONDON, W.C.**

